# Investigating the complex interplay between fibroblast activation protein α-positive cancer associated fibroblasts and the tumor microenvironment in the context of cancer immunotherapy

**DOI:** 10.3389/fimmu.2024.1352632

**Published:** 2024-07-05

**Authors:** Anton Kraxner, Franziska Braun, Wei-Yi Cheng, Tai-Hsien Ou Yang, Shweta Pipaliya, Marta Canamero, Emilia Andersson, Suzana Vega Harring, Sebastian Dziadek, Ann-Marie E. Bröske, Maurizio Ceppi, Tamara Tanos, Volker Teichgräber, Jehad Charo

**Affiliations:** ^1^ Roche Pharma Research and Early Development, Oncology, Roche Innovation Center Basel, F. Hoffmann-La Roche Ltd., Basel, Switzerland; ^2^ Roche Pharma Research and Early Development, Data and Analytics, Roche Innovation Center Munich, Roche Diagnostics GmbH, Penzberg, Germany; ^3^ Roche Pharma Research and Early Development, Data and Analytics, Roche Translational & Clinical Research Center, F. Hoffmann-La Roche Ltd, Little Falls, NJ, United States; ^4^ Roche Pharma Research and Early Development, Data and Analytics, Roche Innovation Center Zurich, Roche Glycart AG, Schlieren, Switzerland; ^5^ Roche Pharma Research and Early Development, Oncology, Roche Innovation Center Munich, Roche Diagnostics GmbH, Penzberg, Germany; ^6^ Roche Pharma Research and Early Development, Oncology, Roche Innovation Center Zurich, Roche Glycart AG, Schlieren, Switzerland

**Keywords:** fibroblast activation protein (FAP), tumor immune microenvironment, T cell infiltration, immune cell subsets, cancer immuno-therapy, patient enrichment

## Abstract

**Introduction:**

This study investigates the role of Fibroblast Activation Protein (FAP)-positive cancer-associated fibroblasts (FAP+CAF) in shaping the tumor immune microenvironment, focusing on its association with immune cell functionality and cytokine expression patterns.

**Methods:**

Utilizing immunohistochemistry, we observed elevated FAP+CAF density in metastatic versus primary renal cell carcinoma (RCC) tumors, with higher FAP+CAF correlating with increased T cell infiltration in RCC, a unique phenomenon illustrating the complex interplay between tumor progression, FAP+CAF density, and immune response.

**Results:**

Analysis of immune cell subsets in FAP+CAF-rich stromal areas further revealed significant correlations between FAP+ stroma and various T cell types, particularly in RCC and non-small cell lung cancer (NSCLC). This was complemented by transcriptomic analyses, expanding the range of stromal and immune cell subsets interrogated, as well as to additional tumor types. This enabled evaluating the association of these subsets with tumor infiltration, tumor vascularization and other components of the tumor microenvironment. Our comprehensive study also encompassed cytokine, angiogenesis, and inflammation gene signatures across different cancer types, revealing heterogeneous cellular composition, cytokine expressions and angiogenic profiles. Through cytokine pathway profiling, we explored the relationship between FAP+CAF density and immune cell states, uncovering potential immunosuppressive circuits that limit anti-tumor activity in tumor-resident immune cells.

**Conclusions:**

These findings underscore the complexity of tumor biology and the necessity for personalized therapeutic and patient enrichment approaches. The insights gathered from FAP+CAF prevalence, immune infiltration, and gene signatures provide valuable perspectives on tumor microenvironments, aiding in future research and clinical strategy development.

## Introduction

In the field of cancer therapeutics, there has been a significant and noteworthy evolution in treatment approaches. The integration of immunotherapeutic agents into the standard-of-care strategies has shown remarkable growth. This trend is observed across a large variety of cancer patients, including those in the early and advanced stages of the disease. This transformative shift highlights the increasing recognition of the immune system’s pivotal role in the management of various cancer indications and across diverse genetic profiles (1). This acknowledgement has reshaped the landscape of cancer treatment, with immunotherapy becoming a vital component of the standard-of-care, offering new hope and prospects for patients facing this formidable disease. An intricate aspect of this landscape revolves around the role played by the tumor microenvironment (TME), specifically the tumor stroma, in shaping the immunotype and immune dynamics within the context of cancer progression. It is evident that the influence of the TME extends well beyond T cells, encompassing cells of the innate immune system, such as monocytes, granulocytes, and natural killer (NK) cells, along with non-immune actors like cancer-associated fibroblasts (CAFs) (2–5). The collective interplay of these diverse cellular components leads to the development of a fibrotic stroma, which paradoxically either suppresses T cell function and impedes their migration into tumor nests, or fosters anti-cancer immunity by generating peri-tumoral lymphoid aggregates or tertiary lymphoid structures (TLSs) in other cases, with the latter being associated with enhanced T cell responses and improved clinical outcomes (6, 7).

However, a recent conceptual breakthrough in this multifaceted interplay sheds light on the crucial role likely played by the fibroblast compartment, particularly the CAFs in TME. These CAFs undergo transformation from normal fibroblasts in response to signals from tumor cells and alterations in oxygen and metabolite gradients, profoundly influencing the structural architecture of the TME (3, 8–11). Among the notable characteristics of CAFs is the expression of fibroblast activation protein α (FAP) (12, 13). Remarkably, FAP expression emerges as a significant prognostic marker in numerous tumor types, often portending unfavorable outcomes, as exemplified by pancreatic ductal cancer and colorectal cancer (CRC) (14, 15). Nevertheless, the relationship between FAP expression and cancer is far from constant. In a study of 112 breast cancer patients, abundant stromal FAP expression correlated with significantly longer disease-free and overall survival, contrasting the trends observed in other cancers (16).

Numerous preclinical and animal studies have previously demonstrated that FAP-expressing stroma is a critical component of the TME that inhibit antitumor immunity (11, 13, 17–19) and depleting FAP+ stroma suppresses tumor growth and restores antitumor immunity (20–25). It is worth noting that a significant body of research investigating FAP and its correlation with tumors has been conducted using transgenic animal models (11, 18, 21), at times involving the use of cells engineered to artificially express FAP (26). Therefore, the findings from these studies call for a judicious and considered interpretation.

In our previous work (27), we investigated the expression of FAP in both primary and metastatic tumor specimens. Our research was dedicated to establishing connections between FAP expression and clinical outcomes within the context of immunotherapy. We assessed how FAP levels in these tumors correlated with patients’ responses to immunotherapeutic and chemotherapeutic interventions, shedding light on the potential significance of FAP as a prognostic marker. In the current investigation, using a sizeable clinical cohort of tumor samples, we have examined the intricate immunological signatures that accompany FAP expression within the TME. Specifically, we have examined the complex interplay between FAP+CAFs and the dynamic landscape of the TME. Understanding the immune response within the context of FAP+CAFs is pivotal, as it not only enhances our comprehension of the TME but also opens new avenues for therapeutic exploration and patient enrichment strategies. By looking into the immune signatures associated with FAP+CAFs and its impact on the broader TME, our study aims to contribute to a more holistic understanding of the interplay between stromal elements, immune responses, and cancer cells.

## Materials and methods

### Ethics statement

All procedures were conducted in accordance with the Helsinki declaration and following ethics approval. Patient participation in clinical trials and specimen acquisition was performed with informed consent. Patient tumor samples included in this study were only used if appropriate informed consent was available. Data were used according to internal processes and guidelines and were analyzed for all treated patients that donated biopsy, regardless of intention-to-treat status.

### Tumor specimens

The tumor specimens were collected from patients with mainly advanced, metastatic disease. Among the different cancer indications, samples from patients with Non-Small Cell Lung Cancer (NSCLC), Head and Neck Squamous Cell Carcinoma (HNSCC), and melanoma had a history of prior treatment with checkpoint inhibitors. In contrast, samples from Pancreatic Ductal Adenocarcinoma (PDAC) and Renal Cell Carcinoma (RCC) patients were checkpoint inhibitor treatment naïve. The majority of specimens were obtained through 16 -18 G core needle biopsies conducted in the context of Roche clinical studies between 2017 and 2021. All samples underwent immediate formalin fixation, processing, and paraffin embedding upon collection.

### Immunohistochemical staining and image analysis

#### Tissue processing

Consecutive 2.5-μm longitudinal sections of formalin-fixed paraffin-embedded tumor tissues were subjected to the following staining procedures as described previously (27). Tissue sections were deparaffinized and rehydrated using standard protocols, followed by staining with specific antibodies as outlined below. After antibody incubations, sections were counterstained with Hematoxylin II for 8 minutes and bluing reagent for 8 minutes. The slides were then dehydrated in ascending alcohol grades, cleared in xylene, and mounted with a cover slip. Digital whole slide images were captured and uploaded onto IRIS (Intelligent Research Imaging Software), our proprietary next-generation digital pathology platform designed for standardized oncological research. Prior to analysis, a board-certified pathologist delineated tumor regions of interest using established internal guidelines. Non-tumor tissue, artifacts, and necrotic regions were manually identified and excluded from the analysis.

#### FAP/KRT assay

Antigen retrieval was carried out using Cell Conditioner 1 (Ventana Medical Systems) for 48 minutes. The tissue sections were then sequentially incubated with two primary antibodies: rabbit anti-FAP antibody (Clone SP325, Spring Biosciences aka Abcam ab227703) for 16 minutes and mouse anti-cytokeratin/KRT antibody (Clone AE1/AE3/PCK26, Ventana Cat. Nr. 05267145001) for 8 minutes, with both incubations maintained at 37°C. Following each primary antibody application, signal detection was performed using the Discovery Purple detection kit for FAP and the Discovery Yellow detection kit for KRT (Ventana Medical Systems).

#### Quantification

The percentage of the tumor area positive for FAP expression was determined using a threshold-based algorithm within IRIS that quantifies the proportion of the area with positive staining relative to the total annotated tumor area. Our methodology ensured the precise detection and quantification of FAP and KRT expression, facilitating reliable downstream analysis of their prognostic significance in tumor tissue samples.

#### CD3/PRF assay

For the CD3/PRF assay to detect cytotoxic T and NK cells, we employed the Ventana Benchmark XT platform using the U IHC DS oDAB-uRed v4 procedure. Tissue sections were pre-treated with Cell Conditioner 1 for 32 minutes and then incubated with the primary mouse antibody to perforin (PRF) (clone 5B10, Abcam Cat. Nr. ab89821) for 12 minutes at 37°C. The bound PRF antibody was detected using the OptiView DAB detection kit (Ventana Medical Systems). Subsequently, after heat denaturation, slides were incubated in the primary rabbit antibody CD3E (clone 2GV6, Ventana Cat. Nr. 05278422001) for 16 minutes at 38°C, and the bound primary antibody was detected using the ultraView Red detection kit (Ventana Medical Systems). Sections were counterstained with Hematoxylin II (Ventana Medical Systems) for 8 minutes, followed by bluing solution for 8 minutes, and then dehydrated and cover-slipped.

#### Ki67/CD8 assay

For the Ki67/CD8 assay to detect CD8 positive either proliferating or non-proliferating, we utilized the Ventana Discovery Ultra platform and the Research Use Only Discovery Universal procedure. Tissue sections were treated with Cell Conditioner 1 for 64 minutes and incubated with the rabbit primary antibody CD8 (clone SP239, Ventana, Cat. Nr. 09780041001) for 32 minutes at 38°C. Bound CD8 antibody was detected using UltraMap anti-rabbit AP secondary antibody and Discovery Yellow detection kit (Ventana Medical Systems). After heat denaturation, slides were incubated in the rabbit primary antibody Ki67 (Clone 30-9, Ventana Cat. Nr. 05278384001) for 8 minutes at 38°C, and the bound primary antibody was detected with Hapten-linked Multimer anti-rabbit HQ and anti-HQ HRP secondary antibody, followed by Discovery Purple detection kit (Ventana Medical Systems). Sections were counterstained with Hematoxylin II for 8 minutes, followed by bluing solution for 8 minutes, and then dehydrated and cover slipped.

#### FoxP3 assay

For the FoxP3 assay to assess T regulatory cells, the Benchmark XT platform, and the XT OptiView DAB IHC v4 procedure were employed. Tissue sections were treated with Cell Conditioner 1 for 32 minutes, followed by incubation with the primary antibody FoxP3 (clone 236A-E7, Abcam Cat. Nr. ab20034) for 60 minutes at 37°C. Positive staining was detected using the OptiView DAB detection kit (Ventana Medical Systems). Sections were counterstained with Hematoxylin II for 8 minutes, followed by bluing solution for 8 minutes, and then dehydrated and cover-slipped.

#### Quantification (immune cells)

The density of immune cells was determined based on a threshold-based algorithm within IRIS that quantifies the number of events with positive staining relative to the total annotated tumor area (events/mm2).

### Quality Control and Reporting

All staining assays included appropriate negative and positive controls. Image analysis involved developing algorithms for detecting and classifying IHC-stained objects on a whole-slide basis using Matlab. After brightfield stain unmixing, IHC-stained objects were identified as cell candidates. These candidates were subjected to the extraction of quantitative features. The classification process, including categorization into various cell classes (e.g., CD8+/Ki67 cells), was accomplished using supervised machine learning, which was trained using a ground truth gallery of true and false stained objects provided by a pathologist. Finally, classified cells and tumor areas, as annotated by a pathologist through digital slide annotation, were reported, and QC images were generated for pathology review. The results of automated digital slide analysis were reported specifically for tumor areas.

### Transcriptomic analysis

#### Bulk RNA sequencing

DNA and RNA co-extraction from formalin-fixed paraffin-embedded (FFPE) samples, followed by the generation of libraries for Illumina TruSeq RNA Access Sequencing, was performed at Q2S/Expression Analysis in the United States, with reference to protocol ID (LAB_13_3256). For the extraction process, core-needle tumor biopsies were utilized, with a requirement of 8 FFPE curls, each measuring 4-5 microns in thickness, totaling 40 microns.

The library preparation phase involved the enrichment of the mRNA fraction through positive selection, achieved by employing a mixture of biotinylated oligos corresponding to the coding regions of the human genome. An essential precondition for proceeding with library preparation was the availability of a minimum input of 100 ng of RNA. Samples not meeting the criterion of %DV200 < 30 were not recommended for further analysis through this method. Following library preparation, the samples were subjected to sequencing using the Illumina sequencing-by-synthesis platform. The sequencing protocol included 50-bp paired-end sequencing, and each sample was subjected to a total read depth of 40 million reads.

Base calling was performed using the BCL to FASTQ file converter, specifically, bcl2fastq v2.17.1.14, available from Illumina’s official website (https://support.illumina.com/downloads.html). For the estimation of gene expression levels, the paired-end RNA-Seq reads were mapped to the human genome (hg38) using the STAR aligner version 2.5.2a, with default mapping parameters as described previously, and the “stranded” option set to “reverse.” To consolidate the number of mapped reads for all Ensembl transcript variants of a given gene (counts), the featureCounts software was employed (28). Subsequently, these counts were normalized as transcripts per million (tpm).

#### Cell type enrichment analysis

To assess cell populations in our tissue samples, cell type enrichment analysis was performed on quality-controlled and normalized transcriptomic datasets. Using the xCell tool, which utilizes gene signatures and ssGSEA as per Aran et al. (29), we calculated enrichment scores to estimate the abundance of various cell types. xCell deconvolution is a computational method that uses gene signatures to infer the abundance of 64 cell types including immune cell types. It is based on a ssGSEA of ~ 10,000 genes and 489 gene signatures extracted from large-scale expression data from six projects (FANTOM, BluePrint, ENCODE, IRIS, HPCA, and Noverstern). This approach is based on ranking of each gene signature in the bulk tissue, then calculating of the enrichment score and spillover compensation to distinguish closely related cell types. For each subset, Spearman correlation coefficients were computed, with significant correlations (false discovery rate, FDR < 0.05) emphasized in bold. This multifaceted approach allowed us to delineate cellular heterogeneity and link it to potential pathogenic mechanisms, providing a comprehensive view of the tissue landscape in relation to disease.

#### Gene signatures

We utilized specific gene signatures of interest, integrating both proprietary Roche internal signatures and publicly available pathway data from REACTOME. The Roche internal signatures included:

Angiogenesis Signatures: comprising genes such as VEGFA, KDR, ESM1, PECAM1, ANGPTL4, and CD34, which are critical for vascular development and remodeling within tumors.

T Effector Signatures: encompassing CD8A, EOMES, PRF1, IFNG, and CD274, which are indicative of cytotoxic T cell activity and immune response modulation.

Myeloid Inflammation Signatures: consisting of IL-6, CXCL1, CXCL2, CXCL3, CXCL8, and PTGS2, representing myeloid cell-mediated inflammatory responses within the tumor microenvironment.

Furthermore, we included gene sets related to cytokine signaling pathways from the REACTOME database (https://reactome.org/ (30);). These pathways were selected based on their relevance to immune modulation in cancer and included:

Interleukin-4/13 signaling (R-HSA-6785807.8)

Signaling by Transforming growth factor-beta family members (R-HSA-9006936)

Interleukin-10 signaling (R-HSA-6783783.5)

Interleukin-6 family signaling (R-HSA-6783589.8)

Interleukin-2 family signaling (R-HSA-451927.7)

Interleukin-1 family signaling (R-HSA-446652.9)

Interferon-gamma signaling (R-HSA-877300)

Interleukin-12 family signaling (R-HSA-447115.7)

Interleukin-17 signaling (R-HSA-448424.8)

Interferon-alpha/beta signaling (R-HSA-909733)

Tumor necrosis factor signaling (R-HSA-75893.10)

Each of these pathways was analyzed to understand their roles in mediating immune responses within the tumor microenvironment, assisting in correlating these molecular signatures with patient clinical outcomes and treatment responses.

### Survival analyses

We subsequently evaluated the prognostic significance of high FAP mRNA expression on the efficacy of cancer immunotherapy. We analyzed transcriptomic and clinical outcome data from clinical trials of Atezolizumab in RCC from the IMmotion151 study and in non-small cell lung cancer (NSCLC) from the Impower131 and Impower150 studies (see table in **Supplementary Information**). Kaplan-Meier survival estimates were generated using the lifelines package in Python. The threshold for high FAP expression was set at the 90th percentile. Additionally, cutoffs for tumor microenvironment (TME) signatures T_EFF (effector T-cells), ANGIO (angiogenesis), and MYELOID (myeloid-derived suppressor cells) were established at the 50th percentile, following the methodology described by McDermott et al (31). Hazard ratios (HR) were computed using Cox proportional hazard regression models, and significance was assessed using Wald test-derived p-values.

### Statistics

To establish the relationships between FAP+CAF content either measured as area coverage or mRNA expression and cell densities (e.g. CD8+ cells) or gene signatures of interest, Spearman’s rank correlation coefficients were computed to assess the monotonic relationships between each identified cell type subset. Significance thresholds were stringently controlled by employing the FDR correction method, with an alpha level of 0.05. This conservative approach was adopted to mitigate the likelihood of Type I errors arising from multiple hypothesis testing, thereby bolstering the veracity of the statistically significant correlations identified in this study. All statistical analyses were carried out using R (Version 4.1.2).

## Results

### FAP content and tumor infiltration is increased in metastasis versus primary tumors in RCC

**Figure 1:**
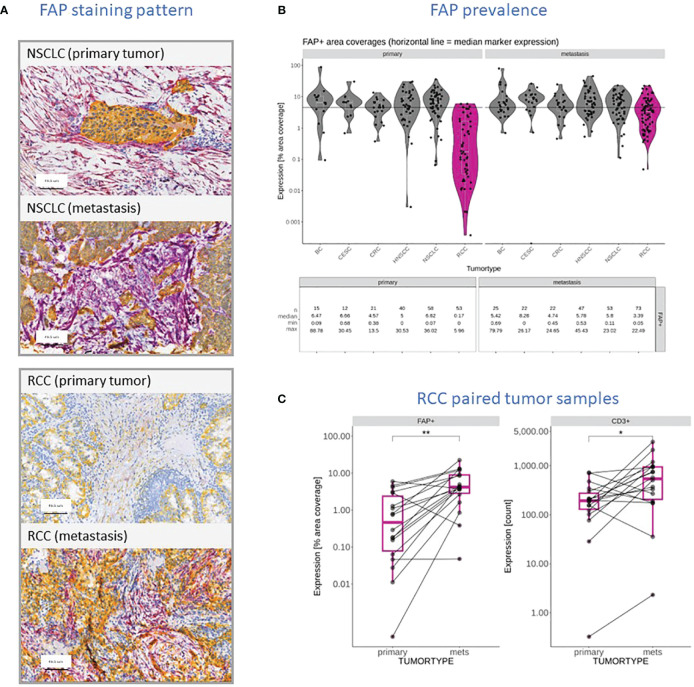
**(A)** Representative FAP (purple) and KRT (yellow) staining pattern obtained through IHC and digital slide analysis from primary and metastatic NSCLC and RCC samples. **(B)** FAP content reported as a percentage of tumor area covered in primary tumors and metastatic lesions within the context of different tumor types, including BC, Squamous Cervical Cancer (CESC), CRC, HNSCC, NSCLC, and RCC (dark pink). The data shows the individual measurements (filed circles), median for each cohort (white line), the overall median value (black line) and summary statistics including the number of samples, the median coverage as well as minimum and maximum values observed. **(C)** Comparison of FAP stained tumor areas and CD3+ T cell infiltration in primary RCC tumors and patient-matched metastatic lesions in a cohort of seventeen patients. Level of statistically significant differences between primary tumors and metastasis indicated as ** (p<0.01) or * (p<0.05).

In our prior work (27), we examined FAP expression across various tumor types and its link with clinical outcomes. In this study, we initially compared FAP+CAF concentration in primary tumors versus metastatic lesions from diverse tumors (**Figure 1A**). While most tumor types showed similar FAP+CAF levels, RCC metastatic samples (n=73) displayed significantly higher FAP+CAF density compared to primary (n=53) tumors (**Figures 1A, B**). Analyzing additional matched RCC samples confirmed this trend (**Figure 1C**). Based on published research (32, 33) and our own observations (27), FAP is commonly found in CAFs, a prevalent cell population in the tumor microenvironment (TME). Interestingly, heightened FAP+CAF content in metastatic RCC coincided with a statistically significant influx of infiltrating T cells (**Figure 1C**). This prompted a closer look into the relationship between FAP+CAF content and the cellular composition of the TME, especially immune infiltration status in FAP-rich tumors.

### Higher FAP content is associated with increased density of tumor infiltrating lymphocytes across tumor types

**Figure 2:**
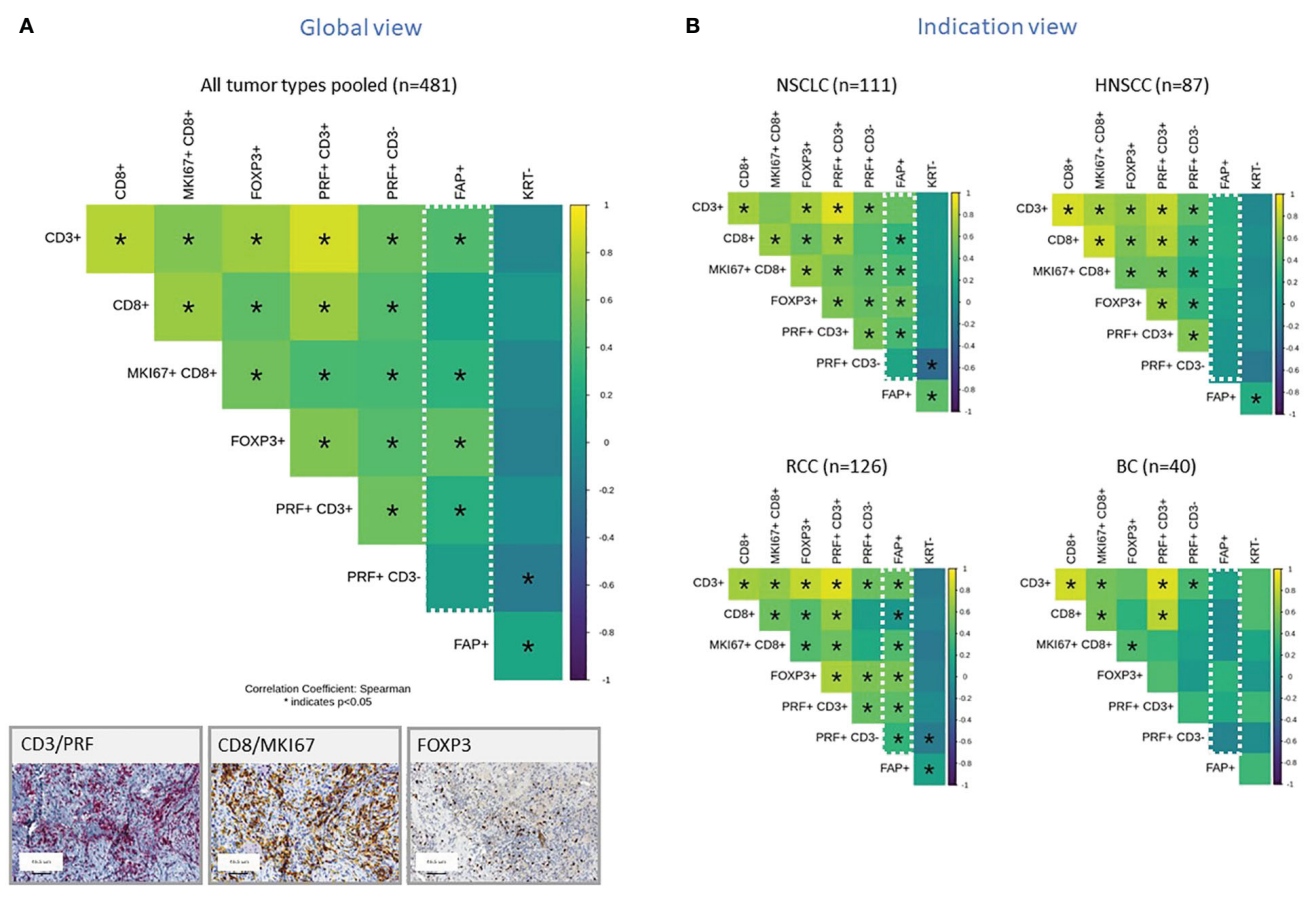
**(A, B)** Correlation Matrix of FAP+ Tumor Stroma Characteristics and Key Immune Cell Populations, Measured by IHC and digital slide analysis. The tumor stroma is defined as KRTnegative, and FAP staining is used to assess FAP-positive tumor stroma. Key immune cell populations are characterized by various markers, as indicated in the legend. Results are presented for all tumor types pooled **(A)** or representative tumor indications **(B)**. The values presented in the matrix correspond to Spearman’s correlation coefficients. Colored scale bar indicates direction and magnitude of correlation. The Spearman correlation coefficient measures the strength and direction of a linear relationship between two variables. When the Spearman correlation coefficient is higher in magnitude (closer to 1 or -1), it indicates a stronger correlation between the two variables. A positive correlation coefficient close to 1 (yellow) suggests a strong positive linear relationship, while a negative correlation coefficient close to -1 (purple) suggests a strong negative linear relationship. On the other hand, a Spearman correlation coefficient close to 0 indicates a weak or no linear relationship between the variables. Asterisk within the squares highlight significant correlations, with significance determined at a level of ¾0.05. Inset panel presents immunohistochemistry staining of immune cells within the tumors. The immune cell populations and stromal characteristics assessed include CD3 T cells (CD3+), CD8 T cells (CD8+), proliferating CD8 T cells (MKI67+CD8+), Tregs (FOXP3+), cytotoxic T cells (PRF+CD3+), NK cells (PRF+CD3-), FAP stained stroma (FAP+), and tumor stroma (KRT-). Representative IHC images are provided in the inset.

In subsequent analyses, we investigated selected subsets of immune cells within the TME using IHC. To do this, we distinguished the non-tumor stromal cell components into two categories: those that were negative for KRT which is the entire stroma excluding tumor cells and those that were specifically positive for FAP. We then constructed a correlation matrix to display the relationships between various immune cell components. The Spearman correlation coefficient, denoted as “ρ,” measures the strength and direction of the nonlinear relationship between two variables. When the Spearman correlation coefficient is close to +1 (yellow), it indicates a strong positive correlation, meaning that as one variable increases, the other tends to increase as well. Conversely, when it is close to -1 (blue), it signifies a strong negative correlation where, as one variable increases, the other tends to decrease. A Spearman correlation coefficient of 0 indicates no correlation between the variables. **Figure 2A** presents the results from 481 tumor samples pooled across the various tumor subtypes. The presence of FAP+ tumor stroma correlated positively with an increased density of CD3+ T cells and FoxP3+ cells which is characteristic of regulatory T cells, as well as proliferating and cytotoxic CD8+ cells. When the analyses were split by individual tumor indications, this correlation was most pronounced in RCC and NSCLC for most of these immune cell markers, while other tumors such as HNSCC and Breast cancer (BC) did not exhibit this significant correlation (**Figure 2B**). Additionally, we observed a general positive correlation of T subsets and NK cells infiltration as well as between FAP+ and KRT- stroma across all indications. The only observed negative correlation was between NK cells (PRF+ CD3-) and KRT- stroma cells.

### Tumors with higher FAP expression are enriched in stromal and immunomodulatory immune cell subsets

**Figure 3:**
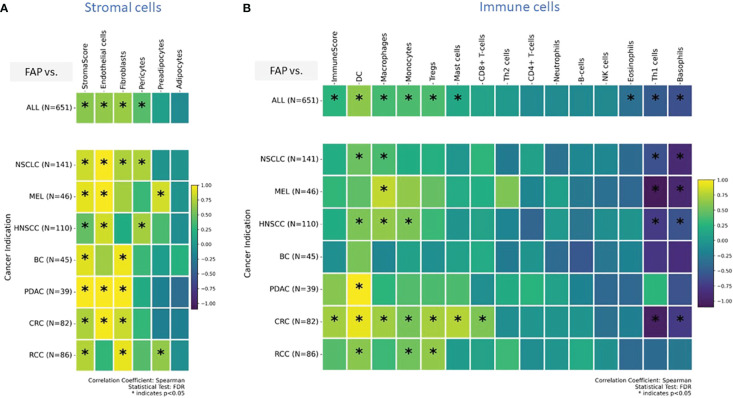
**(A, B)** Enrichment analysis of cell types was conducted on transcriptomic datasets using the xCell gene signatures-based approach, as previously detailed (29). Highlighted are specific stromal and immune cell types in correlation with elevated FAP mRNA expression: a comprehensive view across **(A)** stromal, and **(B)** immune cells (tow row showing on top all tumor indications pooled versus tumor indication specific breakdown in bottom rows). Spearman correlation coefficients were assessed for each subset and plotted. Colored scale bar indicates direction and magnitude of correlation. A positive correlation coefficient close to 1 (yellow) suggests a strong positive linear relationship, while a negative correlation coefficient close to -1 (purple) suggests a strong negative linear relationship. On the other hand, a Spearman correlation coefficient close to 0 indicates a weak or no linear relationship between the variables. Asterisk within the squares highlight significant correlations, with significance determined at a level of FDR ¾0.05. Data from both primary tumors and metastatic lesions were aggregated to increase the overall dataset for each indication. DC - Dendritic cells and MEL, Melanoma.

In a previous publication (27), we have already shown positive correlation between FAP levels measured from IHC or gene expression data. In order to expand our analysis to additional cell types, for which no IHC data was generated, we integrated transcriptomic data, which was generated for the same sample set. In this context, xCell is a useful tool used to gain insights into the cellular composition of tissues or tumors, monitor changes in the immune cell landscape in response to treatments or diseases, and investigate the role of different cell types in various biological processes (29). It is a gene signature-based method used in bioinformatics and genomics to estimate the abundance of different cell types in a heterogeneous tissue sample, typically obtained from bulk RNA sequencing data. This approach deconstructed the cellular makeup of tissues and tumors, revealing the spectrum and relative amounts of various cell types. We used this method to study unique immune and stromal cell subsets in relation to FAP mRNA expression. **Figure 3A** displays results from stromal cells, while **Figure 3B** shows results from immune cells for pooled samples (top row) versus indication specific view (bottom rows).

The “stromal score” (34) gauges both the presence and characteristics of the stromal component within a tumor sample. Its potential applications include prognosis (higher score = worse prognosis), assessment of disease aggressiveness, and predicting therapeutic response. The “immunoscore,” as defined by Galon and Lanzi (35), is a measure of tumor-infiltrating T cells. An elevated immunoscore correlates with better prognosis in various tumors. We used these scores as surrogate predictors for immune response and tumor progression.

The analyses found that in various cancers, there is an increased presence of endothelial cells, fibroblasts, and pericytes. Specifically, in pancreatic (PDAC) and colorectal (CRC) cancers, high FAP levels were linked to more endothelial cells and fibroblasts. This suggests FAP’s role in forming tumor blood vessels and remodeling the surrounding matrix, potentially affecting tumor growth and spread. In contrast, in lung (NSCLC) and head and neck (HNSCC) cancers, high FAP+CAF correlated with more endothelial cells and pericytes, important for blood vessel stability. These findings indicate that FAP+CAFs influence different cancer indications in unique ways, especially in how blood vessels form and function within tumors.

Our analysis of immune cells enrichment showed that, apart from regulatory T cells (Tregs) and CD8+ T cells already identified in IHC based analysis, gene signatures for dendritic cells, monocytes, and macrophages were enriched across tumors. Dendritic cells, monocytes, and Tregs enrichment were most pronounced in CRC and RCC. In contrast, in HNSCC, higher FAP expression was associated with gene signatures for dendritic cells, monocytes, and macrophages but lacked significant enrichment of T cells and Tregs. Notably, a marked decrease in the gene signature associated with T-helper1 cells and basophils was observed across several tumor types, including NSCLC, CRC, HNSCC and melanoma. This suggests a potential downregulation or reduced presence of these immune cells in the TMEs of the mentioned cancer indications (**Figure 3B**).

### Tumors with higher FAP expression are dominated by a TGF- β and Th2-type cytokine signaling network

**Figure 4:**
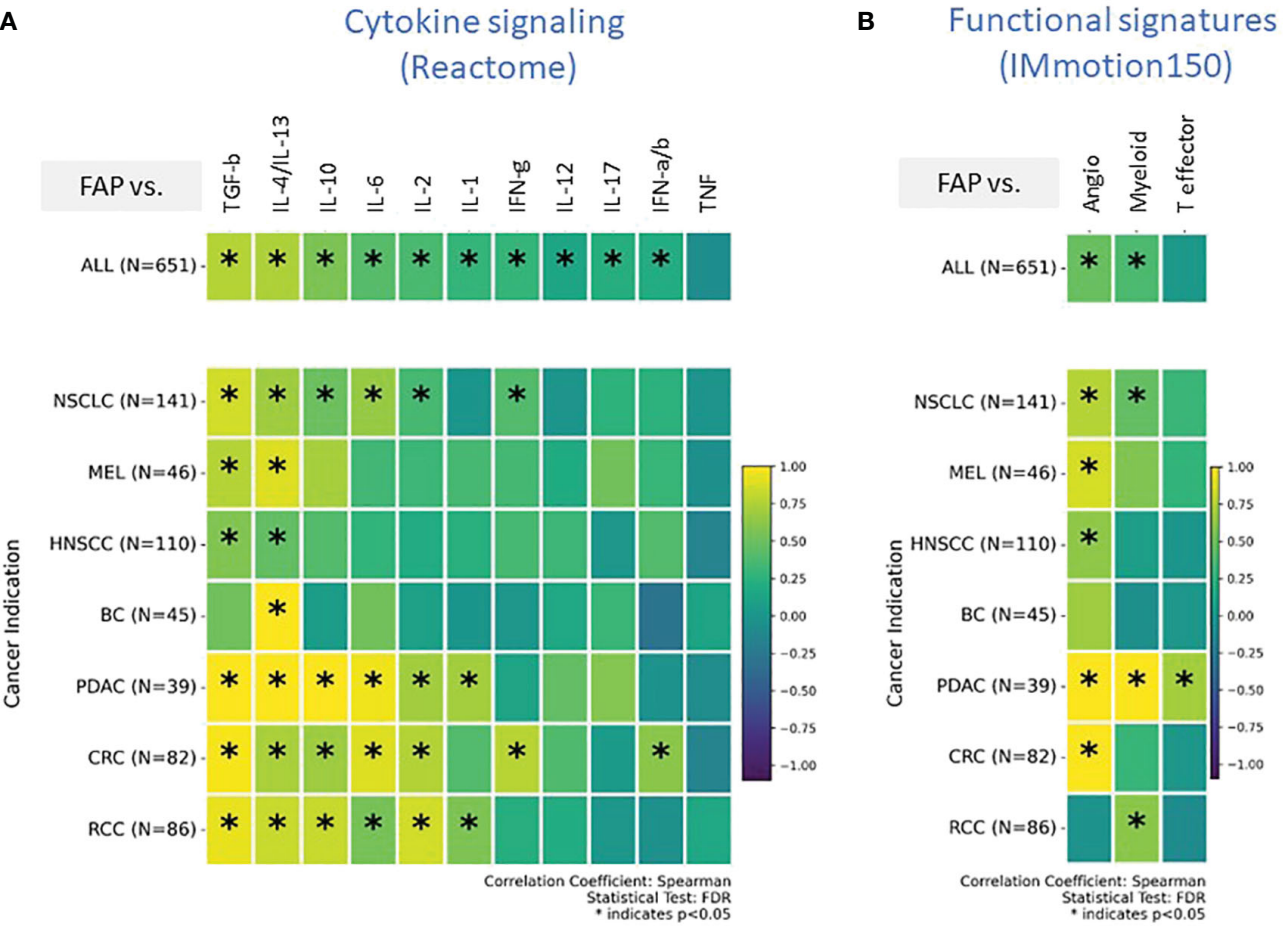
**(A, B)** Comprehensive transcriptomic analysis analyses to evaluate the relationship of FAP mRNA expression with various biological states of the Tumor microenvironment (TME). These states are characterized by distinct gene signature sets, including **(A)** cytokine signaling pathways (sourced from REACTOME database), **(B)** Tumor angiogenesis indicators, infiltration markers, and inflammation indicators (derived from the IMmotion150 study) (31). Spearman correlation coefficients were assessed for each pathway and plotted. Colored scale bar indicate direction and magnitude of correlation. A positive correlation coefficient close to 1 (yellow) suggests a strong positive linear relationship, while a negative correlation coefficient close to -1 (purple) suggests a strong negative linear relationship. On the other hand, a Spearman correlation coefficient close to 0 indicates a weak or no linear relationship between the variables. Asterisk within the squares highlight significant correlations, with significance determined at a level of FDR ¾0.05. Angiogenesis Signatures: VEGFA, KDR, ESM1, PECAM1, ANGPTL4, CD34. T effector Signatures: CD8A, EOMES, PRF1, IFNG, and CD274. Myeloid inflammation Signatures: IL-6, CXCL1, CXCL2, CXCL3, CXCL8, and PTGS2. REACTOME cytokine signaling pathways: Interleukin/IL-1/2/4/6/10/12/13/17, Interferon-alpha/beta/gamma/IFN-a/b/g, Tumor necrosis factor/TNF, Transforming growth factor-beta/TGF-b.

Our transcriptomic analysis effectively outlined the cellular composition but was limited in detailing the immune activation spectrum, specifically in distinguishing between immune-stimulatory and immune-suppressive features. To overcome this limitation and gain insight into cellular functionality, we explored signatures related to cytokine signaling and tumor infiltration and inflammation in subsequent analyses. Our analysis was limited to gene signatures and mRNA quantification due to technical challenges associated with protein measurement in formalin-fixed paraffin-embedded (FFPE) tissues collected during multicenter, multinational clinical trials. Our analysis, depicted in **Figure 4A**, presents data from all tumor types combined, providing insights into commonalities and differences in cytokine pathway activity. We also provide a detailed perspective of individual tumor indications, revealing how each tumor indication expresses a unique cytokine profile.

Both TGF-β and the combined profile of IL-4/IL-13 signaling pathways were found to be consistently upregulated across almost all tumor types. IL-10, IL-6, and IL-2 exhibited upregulated signatures particularly in NSCLC, PDAC, CRC, and RCC. BC was distinct from the rest; the only upregulated cytokine signature in the FAP+ stroma of BC was IL-4/IL-13.

An intriguing aspect of our findings is the absence of enrichment for the “Teff” signature, which has been linked to improved outcomes in response to Atezolizumab (Atezo) and other cancer immunotherapies (CIT) across various tumor types (36, 37) (**Figure 4B**). Specifically, in PDAC, we observed an elevated Teff signature indicative of an active effector T cell response; however, this was juxtaposed with reduced T-helper1 enrichment (**Figure 3B**) and an increased myeloid-driven inflammation, potentially negating the beneficial effects of the Teff cells.

The gene signature indicating angiogenesis (**Figure 4B**), a crucial component for tumor nourishment and growth, was heightened in all tumors. However, BC and RCC did not exhibit an upregulated angiogenesis gene signature associated with FAP expression, potentially suggesting diverse mechanisms of tumor vascularization or alternative nutrient supply routes in these cancer types.

### High FAP expression could be linked to poorer clinical outcomes, even among patient subgroups typically associated with a better prognosis

**Figure 5:**
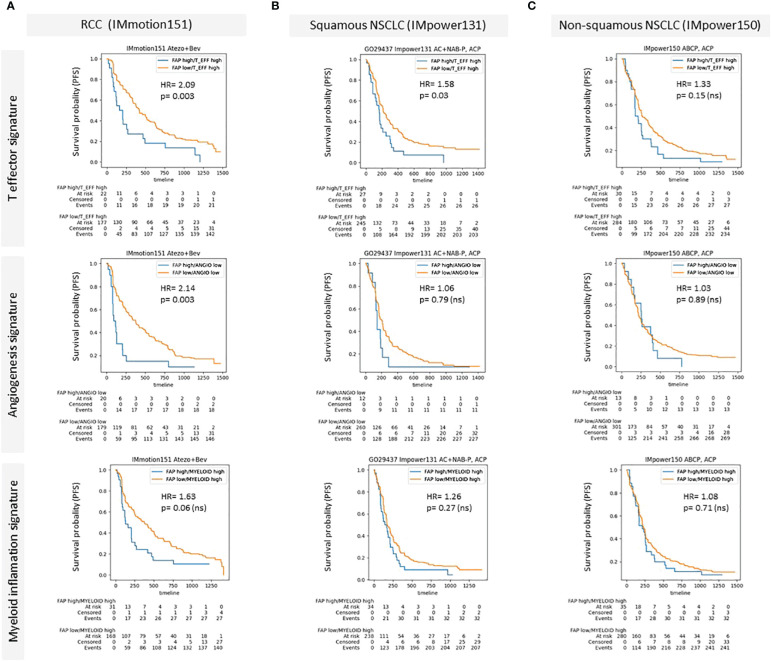
**(A–C)** Kaplan-Meier survival curves demonstrating the impact of high FAP expression on patient outcomes across different cancer types and specific patient subgroups characterized by the presence of prognostic gene signatures (McDermott et al) **(A)** Renal Cell Carcinoma (RCC) data from the IMmotion151 study (Atezo in combination with Bevacizumab), comparing progression free survival rates for high versus low FAP expression across T effector signature, angiogenesis signature, and myeloid inflammation signature. **(B)** Squamous Non-Small Cell Lung Cancer (NSCLC) data from the IMpower131 study (Atezo in combination with chemotherapy) and **(C)** Non-squamous NSCLC data from the IMpower150 study (Atezo in combination with chemotherapy). Each panel displays survival probability (progression-free survival/PFS) on the y-axis against time (months) on the x-axis, with hazard ratios (HR) and p-values provided for each comparison. High FAP expression consistently correlates with poorer outcomes, particularly in RCC and squamous NSCLC across various TME-related pathways. Supplementary: Overview of Roche clinical studies included in this analysis.

We also examined the influence of FAP+CAFs on patient outcomes following immunotherapy in specific patient subgroups. In **Figure 5**, we present Kaplan-Meier survival curves to assess the prognostic impact of FAP+CAFs across different cancer types and pathways within the TME, with previously described prognostic impact (31). This analysis includes data from three clinical studies: IMmotion151, IMpower131, and IMpower150, focusing on RCC, squamous and non-squamous NSCLC, respectively (please refer to table in **Supplementary Information**).

Panel A illustrates the results for RCC (IMmotion151 study), where patients were stratified based on their FAP expression levels and analyzed across three prognostic TME pathways: T effector, angiogenesis, and myeloid inflammation related gene signatures. The data demonstrated a statistically significant negative impact of high FAP expression on progression free survival in the context of T effector high and angiogenesis low signatures, with hazard ratios (HR) indicating substantially worse outcomes (HR = 2.09 and HR = 2.14, respectively) compared to the low FAP subgroup. The relationship in the myeloid inflammation pathway was also negative, though less pronounced (HR = 1.63).

In Panel B, focusing on squamous NSCLC from the IMpower131 study, the impact of FAP was assessed similarly across the same TME pathways. Here, the differences in survival rates were less marked, with statistically significant correlation observed only for T effector high subgroup. The hazard ratios remained around 1 for the other gene signatures, indicating a minimal independent prognostic impact of FAP expression in the other subgroups.

Panel C displays data for non-squamous NSCLC from the IMpower150 study, where similar to squamous NSCLC, high versus low FAP did not show a statistically significant difference in survival outcomes across all examined pathways. The hazard ratios were close to 1, suggesting that FAP expression levels did not substantially influence patient survival in non-squamous NSCLC.

## Discussion

Our preliminary findings brought forth several new learnings: metastatic RCC lesions not only showcased increased FAP+CAF but also displayed a concomitant increase in T cell infiltration when compared to paired primary lesions. This observation supports a temporal and biological relationship between FAP+CAF and immune cell infiltration in paired samples from the same patients. Considering the conventional perception of CAF-dominated tumors reflecting immune-excluded or desert TME, our findings suggest a more complex scenario. We observed evidence of active crosstalk between FAP+CAFs and immune cells within the TME, challenging the conventional view. This interaction appears to be dynamic rather than static, pointing to a situation where CAFs may not generally be synonymous with immune exclusion, but rather the opposite.

The functional consequences of FAP+CAF-immune cell interactions are likely context-dependent, influenced by various factors such as tumor type and immunogenicity, stage, and the host immune status. These nuanced interactions may contribute to the heterogeneity seen in tumor immune responses and potentially impact the efficacy of immunotherapies. It is essential that future studies dissect these complexities to better understand the role of FAP+CAFs in either fostering or inhibiting immune cell infiltration and function within tumors. This prompted us to delve deeper into the correlation between FAP overexpression on one side and the enrichment of major immune cell signatures along with the activation of metabolic pathways on the other side across an array of cancer types.

Thorough immunohistochemical and transcriptomic analyses revealed significant heterogeneity in the TME linked to increased FAP+CAF. This discovery underscores the need for biomarker stratified therapeutic strategies given the variability in both the type and extent of cellular infiltration.

Our analysis was confined to examining gene signatures and quantifying mRNA levels due to the technical difficulties inherent in measuring proteins within formalin-fixed paraffin-embedded (FFPE) tissues, which were collected from multicenter, multinational clinical trials. The challenges in protein measurement from FFPE samples arise from the fixation process, which can degrade or mask proteins, making reliable detection problematic. Nevertheless, the association between the upregulation of cytokines TGF-β, IL-4/IL-13, IL-10, IL-6, and IL-2 and the overexpression of FAP across multiple tumor types highlights a crucial link between FAP+ CAFs, tumor progression, and evasion of immune surveillance. This finding underscores the complex interplay within the tumor microenvironment, significantly influencing cancer development and progression.

FAP is a transcriptional target of TGF-β (38). As such, it was reassuring to see the clear association between TGF-β and FAP+CAF enrichment in our analysis. TGF-β is known for its role in immunosuppression, promoting epithelial-to-mesenchymal transition, and supporting angiogenesis. Its upregulation in FAP+ stroma may contribute to creating an immunosuppressive TME that protects tumor cells from immune-mediated destruction (39)

The expression of IL-4/IL-13 underscores their role in the potential alternative activation of macrophages, known as M2 polarization. M2 macrophages are characterized by their tumor-promoting functions, including support for angiogenesis, tissue remodeling, and suppression of cytotoxic T cell responses. Their activation can directly benefit tumor cells by fostering a pro-tumoral niche (40).

IL-10’s role in cancer is complex, evidenced by its contribution to both immune suppression and stimulation within the FAP+ stromal compartments of diverse tumors such as NSCLC, PDAC, CRC, and RCC. Traditionally viewed as an immune suppressant, IL-10 curbs TH1 and cytotoxic T-cell responses (41, 42), promoting an immunosuppressive microenvironment conducive to tumor evasion. Yet, it also can enhance CD8+ T cell and NK cell activity, especially in conjunction with IL-2 (43), and manipulate tumor surveillance, as seen in both IL-10 deficient and IL-10 overexpressing murine models (44).

Similarly, IL-2, generally known for its role in T and NK cell activation and proliferation, is also required to maintain and expand Tregs (45, 46). It is also notable that FAP+CAF increase coincided in NSCLC and CRC with the enrichment of IFN-. Altogether, FAP overexpression was associated with an active and dynamic cytokine signaling in the TME. Nevertheless, general interrogation of the cytokine bias in a TME associated with FAP overexpression suggested that, apart from PDAC, there was a consistent Th1 biased TME in all studied indications.

A limitation of the current analysis is the inability to measure FAP catalytic activity, which is a crucial aspect of its function. This could have been done using frozen tissues (47), but our samples were composed of formalin-fixed, paraffin-embedded (FFPE) tissue. Consequently, we were unable to assess any association between FAP catalytic activity and TME composition or clinical outcomes.

The results of the Kaplan-Meir curves presented in **Figure 5** suggest that high density of FAP+CAFs in the tumor milieu is associated with poorer clinical outcomes, particularly in RCC, and indicate the potential of FAP as a prognostic biomarker in certain cancer types and TME contexts. The variability in the impact of FAP+CAF content across different cancers and TME pathways underscores the complexity of the tumor microenvironment and the need for a tailored approach in the use of FAP as a therapeutic target and as a biomarker to inform patient enrichment strategies.

Collectively, these findings reinforce the significance of FAP+CAF as pivotal player in the TME. FAP association with a myriad of cytokines that modulate both tumor behavior and the immune response underscores the necessity for a deeper understanding of its function. Modulating FAP expressing stromal cells or their associated cytokine networks may offer novel therapeutic avenues for enhancing the efficacy of current treatments and potentially reversing immunosuppression in TME.

## Data availability statement

Due to Roche company policy and patient privacy reasons access to individual patient level data is restricted. Qualified researchers may request access to individual patient-level data through the clinical study data request platform (RRID:SCR_018080, https://vivli.org/). Further details on Roche's criteriafor eligible studies are available here (https://vivli.org/ourmember/roche/). For further details on Roche's Global Policy on the Sharing of Clinical Information and how to request access to related clinical study documents, see here (https://www.roche.com/innovation/process/clinical-trials/data-sharing/).

## Ethics statement

All procedures were conducted in accordance with the Helsinki declaration and following ethics approval. Patient participation in clinical trials and specimen acquisition was performed with informed consent. Patient tumor samples included in this study were only used if appropriate informed consent was available. Data were used according to internal processes and guidelines and were analyzed for all treated patients that donated biopsy, regardless of Intention-to-treat status. A list of involved ethic committees/review boards is provided in the **Supplementary Material**.

## Author contributions

AK: Conceptualization, Methodology, Visualization, Writing – original draft, Writing – review & editing. FB: Conceptualization, Data curation, Formal Analysis, Methodology, Visualization, Writing – review & editing. W-YC: Conceptualization, Data curation, Methodology, Visualization, Writing – review & editing. T-HY: Conceptualization, Data curation, Formal Analysis, Methodology, Visualization, Writing – review & editing. SP: Formal Analysis, Writing – review & editing. MC: Conceptualization, Methodology, Writing – review & editing. EA: Conceptualization, Methodology, Writing – review & editing. SH: Conceptualization, Methodology, Writing – review & editing. SD: Conceptualization, Writing – review & editing. A-MB: Writing – review & editing. MC: Writing – review & editing. TT: Writing – review & editing. VT: Writing – review & editing. JC: Conceptualization, Methodology, Writing – review & editing.
