# Supplementary figures and images for: Investigating the complex interplay between fibroblast activation protein α-positive cancer associated fibroblasts and the tumor microenvironment in the context of cancer immunotherapy
Source: Front Immunol. 2024 Jul 5;15:1352632. doi: 10.3389/fimmu.2024.1352632 (PMC11258004; doi:10.3389/fimmu.2024.1352632)

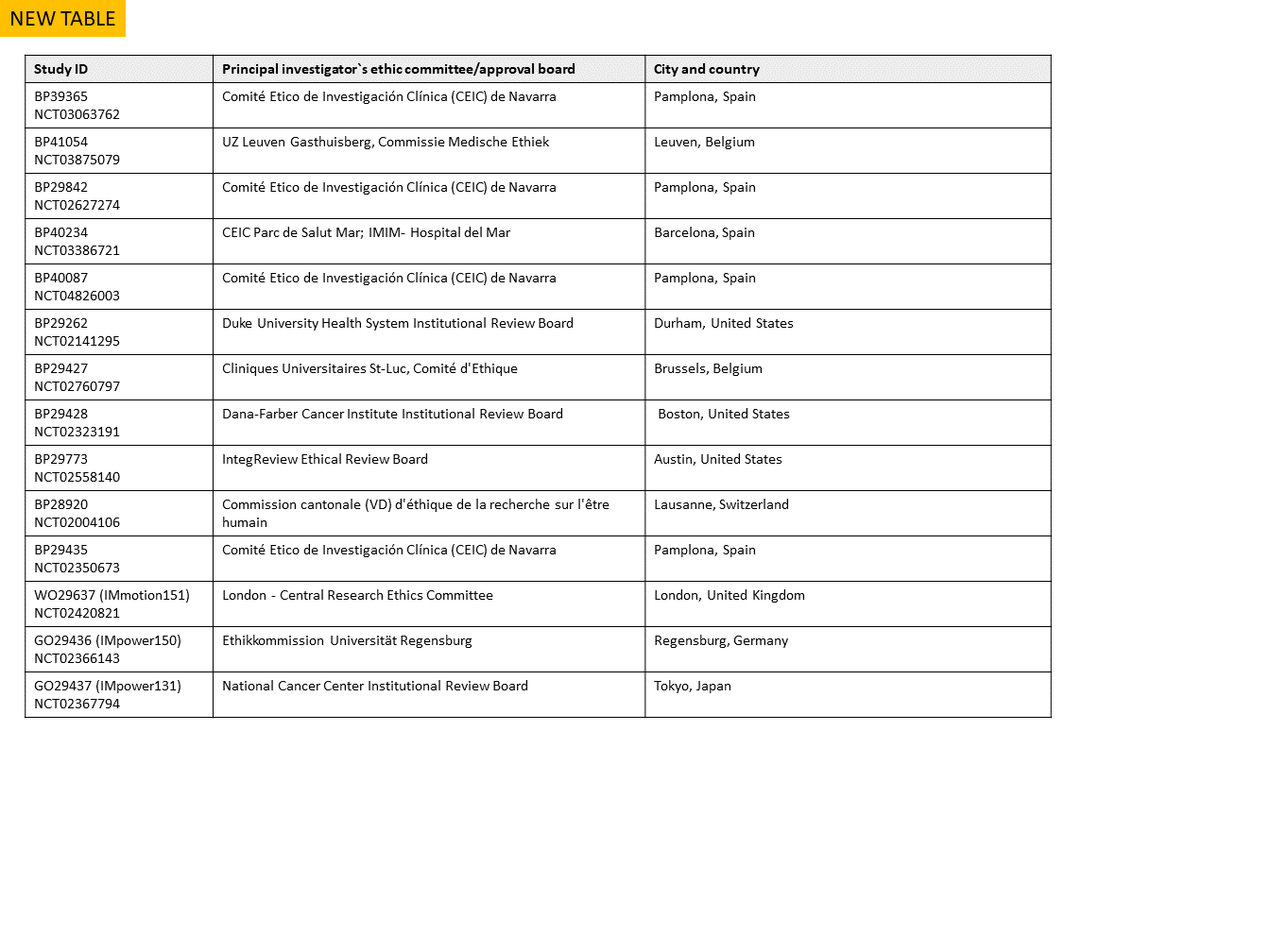

Supplement: Supplementary file 1 [file Image_1.png]
